# Chemosaturation with percutaneous hepatic perfusion of melphalan for liver-dominant metastatic uveal melanoma: a single center experience

**DOI:** 10.1186/s40644-019-0218-4

**Published:** 2019-05-30

**Authors:** Christoph Artzner, Oliver Mossakowski, Gerald Hefferman, Ulrich Grosse, Rüdiger Hoffmann, Andrea Forschner, Thomas Eigentler, Roland Syha, Gerd Grözinger

**Affiliations:** 1 Department of Diagnostic and Interventional Radiology, University Hospital of Tübingen, Hoppe-Seyler-Strasse 3, 72076 Tübingen, Germany; 2 The Warren Alpert Medical School of Brown University, Providence, RI USA; 3 Department of Dermatology, University Hospital of Tübingen, Hoppe-Seyler-Strasse 3, 72076 Tübingen, Germany

**Keywords:** Ocular melanoma; percutaneous hepatic perfusion, Chemosaturation, Liver;

## Abstract

**Objective:**

To investigate the outcome and safety data of chemosaturation with percutaneous hepatic perfusion (CS-PHP) of melphalan in patients with liver-dominant metastatic uveal melanoma.

**Material and methods:**

This is a HIPAA compliant, IRB approved, retrospective study. A total of 28 CS-PHPs were performed in 16 individual patients (six men and ten women, median age 63.1 years [range 49.1 to 78.7 years], one to six CS-PHP procedures per patient) for treatment of liver-dominant metastatic uveal melanoma between June, 2015 and December, 2018. All patients received cross-sectional imaging at baseline and during follow-up. CS-PHP was performed with the Hepatic CHEMOSAT® Delivery System (Delcath Systems, Inc., NY, USA) facilitating extracorporeal filtration of hepatic blood for melphalan removal. Ideal body weight-adjusted melphalan doses were administered into the hepatic arteries. Serious adverse events (SAE), progression-free survival based on response criteria in solid tumors, and overall survival were noted. Survival data were analyzed using Kaplan-Meier estimates.

**Results:**

Partial response after first CS-PHP was observed in nine patients (60%), stable disease in five patients (33%) and progressive disease in one patient (7%). Median overall survival was 27.4 months (95% CI 4.1 to 35.4 month) after first CS-PHP. Median progression-free survival was 11.1 months after first CS-PHP (95% CI 4.9 to 23.6 months). SAEs were observed in the majority of patients with most SAEs limited to grades one and two. Thirteen SAEs of grades three and four were observed in seven individual patients. No grade five SAE was observed.

**Conclusion:**

CS-PHP is an efficacious and safe treatment for patients presenting with liver-dominant metastatic uveal melanoma.

## Introduction

Uveal melanoma is the most common primary intraocular malignancy [1]. The literature reports that about 20–30% of patients with primary uveal melanoma die of systemic metastases within 5 years of diagnosis, a figure that rises to 45% within 15 years [2, 3]. Outcomes for patients with systemic disease are generally poor, with a median overall survival (OS) ranging from 4 to 15 months [1, 3–5]. A recent meta-analysis of 29 phase II trials in metastatic uveal melanoma between 1988 and 2015 with the aim to define historical benchmarks of progression-free survival (PFS) and OS found disappointing outcomes across all treatment groups, with a median PFS of 3.3 months (6-month PFS 27%) and median OS of 10.2 months (1-year OS 43%) [1, 6].

The most frequently affected organ for distant metastases of uveal melanoma is the liver. An analysis of 435 patients included in the Collaborative Ocular Melanoma Study found that 93% of patients had liver metastases at the time of death [7]. Of those who had only one site of metastasis, the liver was involved in 95% of cases. In a small subset of select cases, resection of hepatic lesions may offer enhanced long-term survival; however, very few cases are likely to benefit from resection overall [8].

As a consequence of this strong predilection for hepatic involvement, liver-directed therapies represent an important research focus in the treatment of metastatic disease. A range of endovascular therapies are presently available for the treatment of both primary and metastatic hepatic malignancy, including bland arterial embolization, chemoembolization using a variety of chemotherapy agents (e.g., fotemustine, BCNU, cisplatin), radioembolization using yttrium-90-labeled microspheres, and immunoembolization using granulocyte-macrophage-colony-stimulating factor (GM-CSF) [9–12]. Chemosaturation with percutaneous hepatic perfusion (CS-PHP) represents a more recent minimally invasive and repeatable targeted hepatic therapy. In CS-PHP, melphalan is directly delivered to the hepatic artery; venous blood from the liver is then recirculated through an extracorporeal filtration system, which removes the melphalan before returning the blood to systemic circulation. By utilizing this method, high doses of melphalan are directed to the liver while minimizing systemic exposure [13, 14].

In hepatic metastases of uveal melanoma, CS-PHP with melphalan has been reported to have response rates of up to 83% as well as improved local tumor control [8, 14, 15]. Recently, a large trial with 93 enrolled patients additionally reported superior rates of hepatic progression-free survival (hPFS) in patients with liver-predominant ocular or cutaneous melanoma treated with CS-PHP compared to best alternative care (arterial embolization, systemic chemotherapy, and symptomatic care via supportive measure only). However, the recruitment period was between 2006 and 2009; as a result, all cases included in this work utilized an older filter system that has since been updated in 2012 to a new generation system [16], which has mainly been evaluated with aspects to safety yet [17, 18]. As a consequence, the need exists for updated data regarding efficacy of the most recent CS-PHP technique for the treatment of hepatic metastases of uveal melanoma and supplement data regarding safety.

This retrospective, single-center study meets this need by reporting the outcome and safety data of CS-PHP of melphalan in a cohort of patients with metastatic uveal melanoma with hepatic involvement. In contrast to prior studies that included patients with either various histological entities or without extrahepatic disease, this retrospective study investigates the effects of treatment for a real-world cohort with liver-dominant metastatic uveal melanoma.

## Material and methods

This is a HIPAA compliant, IRB approved, retrospective study with waiver of informed consent. Between June, 2015 and September, 2018, 16 consecutive patients (ten female and six male patients) with unresectable hepatic metastases of uveal melanoma underwent 28 CS-PHP procedures with melphalan. Patients were selected for CS-PHP according to the decision of the interdisciplinary tumor board at the lead authors’ home institution. Median age at first CS-PHP was 63.1 years (range 49.1 to 78.7 years) and a median BMI of 26.0 kg/m^2^ (range 20.6 to 35.3 kg/m^2^). Patient characteristics are summarized in Table 1.

**Table 1:** Patient characteristics

	All	Male	Female
Age (years)	63.1 (49.1–78.7)	65.0 (56.9–75.8)	61.6 (49.1–78.7)
Weight (kg)	75.5 (57–102)	81 (65–96)	71.5 (57–102)
Height (cm)	167 (154–192)	174 (167–192)	164 (154–178)
BMI (kg/m^2^)	26.0 (20.6–35.3)	25.6 (20.6–34.4)	26.1 (21.7–35.3)
BSA (m^2^)	1.88 (1.59–2.19)	2.04 (1.75–2.07)	1.80 (1.59–2.19)
Dose of melphalan (mg)	183 (150–220)	206 (192–220)	171 (150–199)

Of note: all demographic data referred to the time of the first CS-PHP. All values were given as the median with the range in parenthesis. *BMI* Body-Mass-Index. *BSA* Body Surface Area

### Baseline and follow-up cross-sectional imaging

Baseline cross-sectional imaging (both CT and MRI) was obtained before CS-PHP for all patients. CT data were acquired using a 128-row detector multislice CT with non-enhanced, arterial, and portal venous contrast media phases. MRI data consisted of a minimum of a T2 weighted sequence, a non-enhanced T1 weighted sequence, and three dynamic contrast-enhanced T1 weighted sequences obtained via state-of-the-art MRI scanners at 1.5 or 3 Tesla (Siemens Aera and Siemens Skyra, Erlangen, Germany). The median interval between baseline assessment and CS-PHP was eight days (interquartile range 1 to 14 days). Follow-up cross-sectional imaging consisted of liver MRI and whole-body CT for all patients. Follow-up cross-sectional imaging was scheduled every 3 months. The median interval between CS-PHP and follow-up imaging was 81 days (interquartile range 50 to 94 days).

### Image assessment

Image assessment was conducted during a joint reading session by two radiologists in consensus. Both radiologists had expertise in abdominal and oncologic imaging, with eight and 10 years of experience, respectively. The extent of disease was noted, and the response to therapy was characterized using Response Evaluation Criteria In Solid Tumors (RECIST 1.1) [19]. Readers were not blinded to clinical data. The reads did not disagree in any of the cases regarding tumor response.

### Preprocedural therapies and extent of disease

All patients were diagnosed with liver-dominant metastatic uveal melanoma. The median time between the initial melanoma diagnosis and detection of hepatic metastases was 2.4 years (interquartile range 0 to 3.9 years). All patients had metastatic lesions in both lobes of the liver. The number of hepatic lesions per patient ranged from three to more than 20 (fewer than ten lesions in seven patients, more than ten lesions in eight patients, and more than 20 in one patient) corresponding to a median tumor load of 22.5% (interquartile range 10 to 25%). The tumor load was determined as a visual estimate by the readers as CS_PHP is recommended for patients with less than 50% tumor load of the liver by the manufacturer.

Eight patients (50%) presented with additional extrahepatic metastases before their first CS-PHP. The most common sites of extrahepatic metastases were the bones (five patients), lungs (four patients), lymph nodes (one patient), and spleen (one patient). Six patients had received systemic chemotherapy using the immune checkpoint inhibitors ipilimumab and nivolumab (five patients) and pembrolizumab (one patient), which had been discontinued prior to baseline imaging and subsequent first CS-PHP procedure. Alternative local therapies targeting liver lesions had been additionally performed in four patients, with three patients had received radiofrequency ablation of single metastasis and one patient had undergone surgical resection of a single metastasis. While patients were treated with CS-PHP, immunotherapy was discontinued. One patient underwent surgery for resection of an adrenal metastasis after first CS-PHP. Two patients were treated with Carboplatin and Paclitaxel and three patients received nivolumab after progressive disease was diagnosed following their last therapy with CS-PHP.

### Pre-procedural preparation and requirements

Embolization of selected arterial branches supplying the gastrointestinal tract was performed as needed to avoid inadvertent extrahepatic administration of chemotherapy to the gastrointestinal or other visceral arterial branches. Lab work requirements were platelet counts of more than 50,000 per mL, INR less than 1.6; glomerular filtration rate of more than 30 mL/min/1.73 m2; no severely impaired liver function based on bilirubin and albumin.

### Treatment

Patients received melphalan delivered using the Hepatic CHEMOSAT® Delivery System (Delcath Systems, Inc., NY, USA) via the manufacturer’s recommendations, which have been described in detail in previous literature [14]. The median time between diagnosis of hepatic metastases and first CS-PHP administration was 4.7 months (interquartile range 2.2 to 10.4 months). The procedure was performed under general anesthesia in an interventional radiology suite. Percutaneous venous and arterial access were obtained using ultrasound guidance to minimize both the number of puncture-attempts and bleeding risk. Access routes were (1) a four French left common femoral artery access used to place a microcatheter in the hepatic arteries for administration of melphalan; (2) a ten French right jugular vein access used to return the filtered blood; and (3) a twelve French right common femoral vein access used to place a double balloon catheter for venous liver isolation. Heparin (400 IU/kg body weight) was administered before initiation of extracorporeal filtration to avoid clotting and was monitored peri-procedurally via activated clotting time (ACT) with a target ACT of greater than 450 s. Of note, despite all patients were prepared by administration of large quantities of fluids before establishment of the extracorporeal hemofiltration circuit, transient hypotension was observed in all patients after inflation of the occlusion balloons and establishment of the extracorporeal hemofiltration circuit. After hemodynamic stabilization was achieved by administartion of additional fluids and vasopressors, melphalan was administered at a dose of 3.0 mg/kg ideal body weight (maximum dose 220 mg/treatment session). The melphalan dose was dissolved in 530 mL of 0.9% NaCl and administered at a flow rate of 0.4 mL/s. Blood flow was monitored after each administration of 100 mL of dissolved melphalan solution by angiography; potential flow limitations by means of visible vasospasms were treated with intraarterial administration of nitroglycerin in 11 of 28 procedures whereas liver arteries originating from the mesenteric artery seemed to be especially prone to vasospasms. Venous hepatic blood was filtered for melphalan removal for an additional 30 min after the infusion of melphalan was ended. Protamine was administered after hemofiltration was stopped. Access sheathes were removed after stabilization of activated clotting time, whereas the arterial access sheath was removed directly after the procedure using a closure device (Femoseal, Terumo Europe NV, Leuven, Belgium). Venous access sheaths were removed on the intensive care unit within 24 h. The median total procedure time was 3.5 h (range, 2.9 to 4.1 h). All patients stayed on intensive care unit for the first 24 h after the treatment. Some patients received more than one CS-PHP, based on there initial response, the remaining tumor of the liver, and the clinical course of the disease.

### Serious adverse events

Serious adverse events (SAE) were categorized using the Common Terminology Criteria for Adverse Events (CTCAE) Version 5.0 (2017). Follow-up for the occurrence of SAEs was conducted using the electronic medical records clinical information system of the authors’ home institution and was based on discharge documents, laboratory records, and associated documentation reports. Median follow up regarding SAEs based on lab work and patient reports was 16 days (range 3 to 42 days).

### Statistical analysis

All data were reported as median and either total range or interquartile range. Kaplan-Meier estimators were used as non-parametric statistics to approximate the survival function. Correlations were calculated using Spearman’s ρ. *P*-values of α < 0.05 were regarded as statistically significant. Statistical analysis was performed using JMP 14.2 (SAS Institute Inc., Cary, NC, 1989–2019) and SPSS 25 (IBM Cooperation, Armonk, USA.

## Results

### Tumor response

**Fig. 1:**
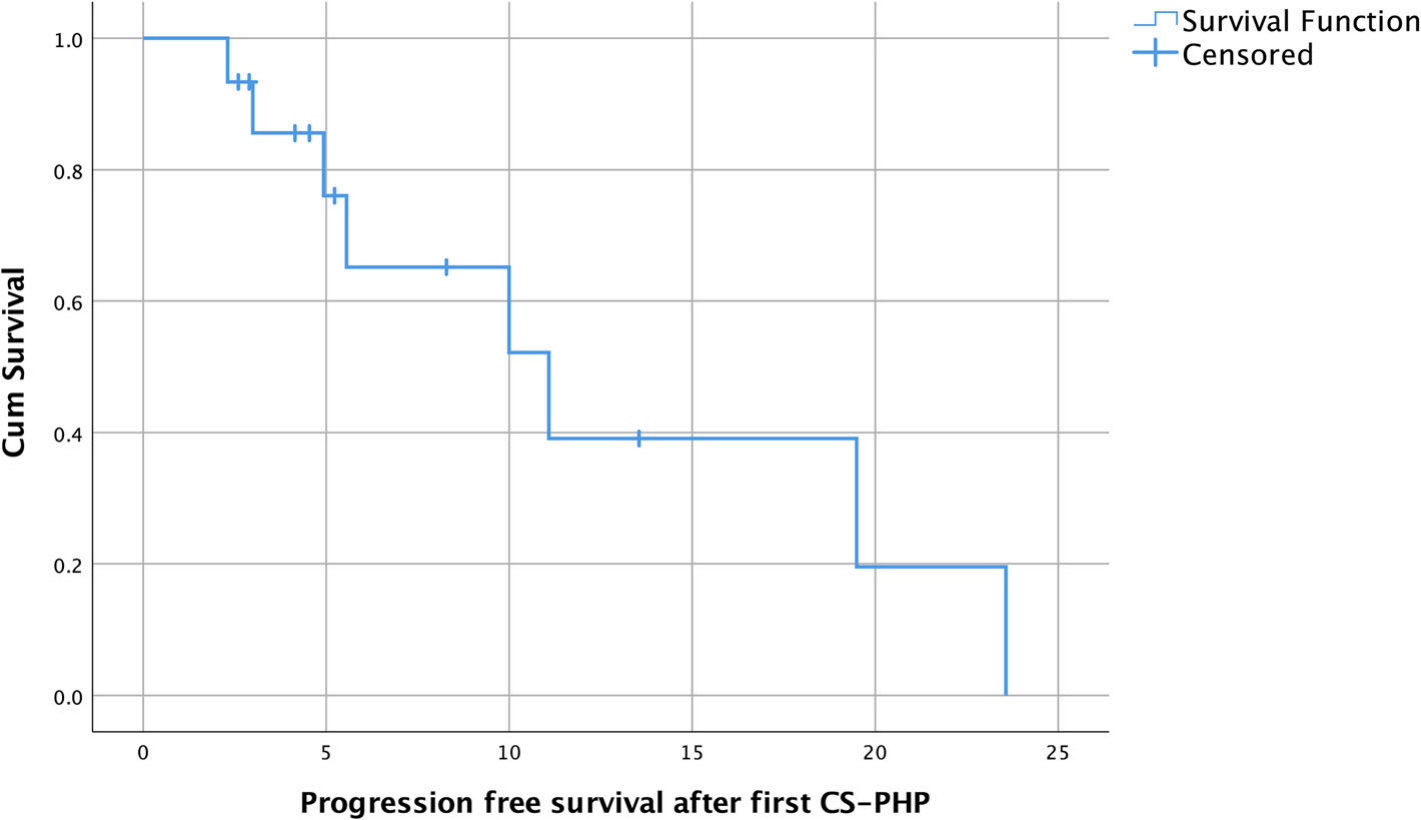
Progression-free survival Kaplan Meier estimates. Of note: three patients received a second chemosaturation therapy without evidence of progressive disease

Partial response (PR) after first CS-PHP was observed in nine patients (60%), stable disease (SD) in five patients (33%) and progressive disease (PD) in one patient (7%). One patient was removed from the study during first CS-PHP treatment due to intra-procedural cardiac arrest (please see description in the serious adverse events section). Three patients received a second CS-PHP treatment before showing progressive disease. Progression-free survival after first CS-PHP was 11.1 months (95% CI 4.9 to 23.6 months; quartile survival times [25 and 75%] 19.5 and 5.6 months; Fig. 1).

Six patients underwent a second CS-PHP session with PR in four patients (67%), and SD in two patients (33%). Progression-free survival after second CS-PHP was 9.6 months (95% CI 7.0 to 19.76 months; quartile survival times [25 and 75%] 7.4 and 15.6 months).

Three patients received a third CS-PHP, resulting in SD in all three patients. One patient received a fourth, fifth, and sixth CS-PHP that resulted in SD, SD, and PD responses, respectively.

**Fig. 2:**
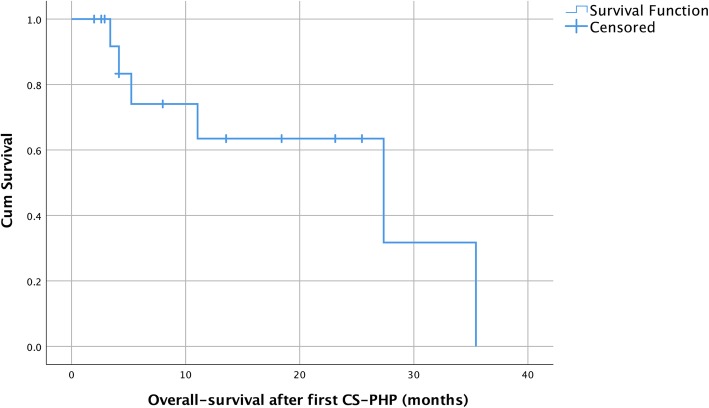
Overall survival Kaplan Meier estimates. Of note: 15 individual patients were treated with 28 chemosaturation therapies

**Fig. 3:**
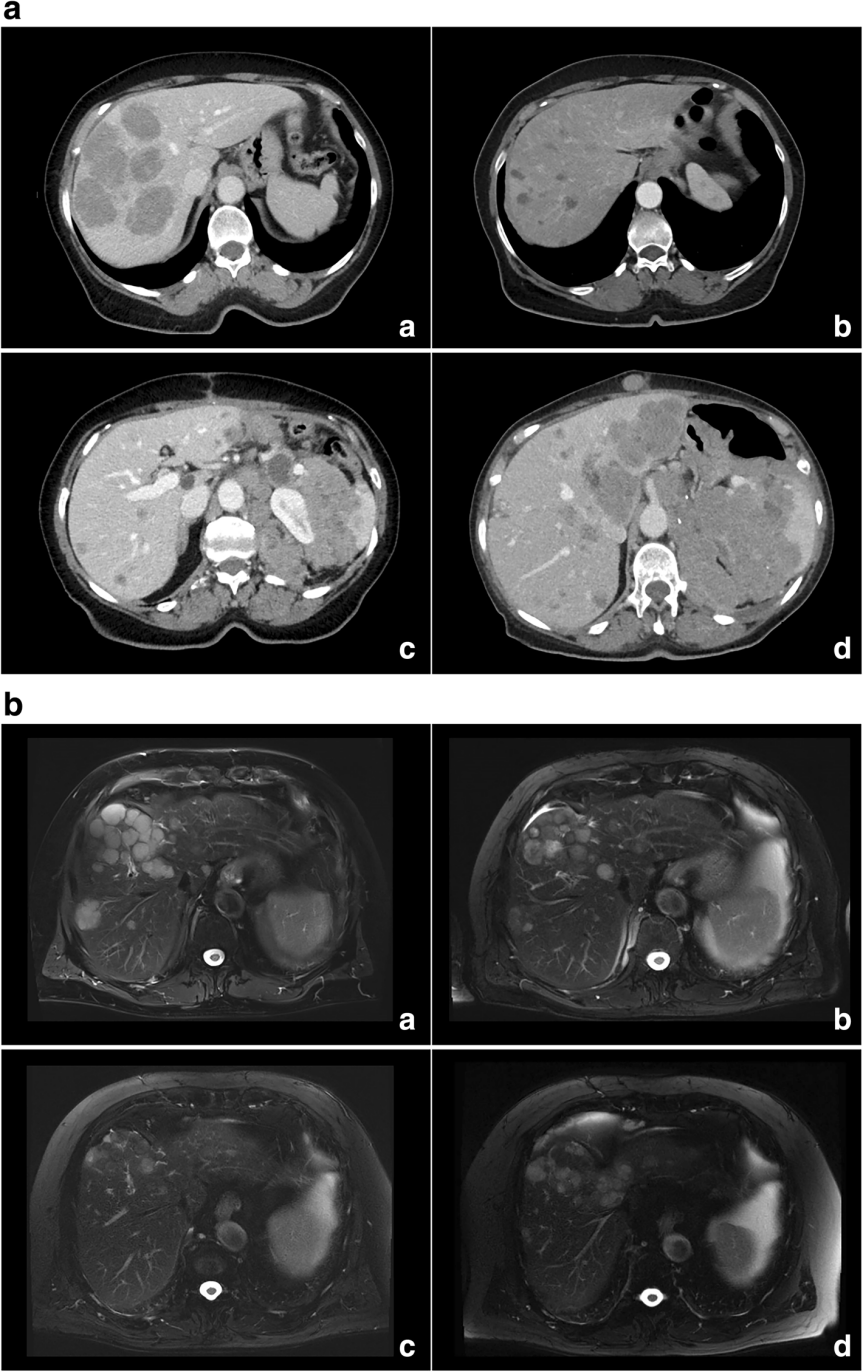
**a**: Patient case. Of note, **a** depicts a patient with liver dominant metastatic uveal melanoma at baseline before first chemosaturation with percutaneous hepatic perfusion (CS-PHP) treatment. The patient responded with partial remission 3 month after first CS-PHP procedure (**b**). Sustained hepatic tumor control was achieved for 30 months by five treatments with CS-PHP, while extrahepatic progressive disease was noted (**c**). After a sixth CS-PHP application and 35 months after first CS-PHP therapy, progression was diagnosed intra- and extrahepatically (**d**). **b**: Patient case. Of note, (**a**) T2 weighted images of a patient with liver dominant metastatic uveal melanoma at baseline before first chemosaturation with percutaneous hepatic perfusion (CS-PHP) treatment. The patient responded with partial remission 3 month after first CS-PHP procedure (**b**). Sustained hepatic tumor control was achieved for 13 months (**c**). Progression was diagnosed intrahepatically after 17 months, and patient was scheduled for a new CS-PHP (**d**)

Median overall survival was 27.4 months (95% CI 4.1 to 35.4 months; quartile survival times [25 and 75%] 35.4 and 5.2 months; Fig. 2). Figure 3 illustrates the typical course of a patient’s hepatic disease. One-year survival was 58%. Median follow-up was 6.13 months (interquartile range 2.8 to 20.4 months).

Survival estimates were grouped by liver-dominant and liver-limited metastatic disease before first CS-PHP. In these subgroups, OS was 27.4 months (95% CI 3.4 to 27.4 months) and 35.4 months (95% CI 4.1 to 35.4 months) for liver-dominant and liver-limited disease, respectively. However, these differences were not statistically significant due to a small number of subjects in each subgroup.

A weak correlation was found between PFS after first CS-PHP and tumor load of the liver before first therapy session (Spearman’s ρ = − 0.52; ρ^2^ = 0.27; *p* < 0.05). The correlation of tumor load and OS was not significant (Spearman’s ρ = − 0.70; ρ^2^ = 0.49; *p* = 0,12), potentially due to fewer data points.

### Serious adverse events

SAEs from categories one to four were observed after CS-PHP. These include leukopenia (96%), anemia (96%), thrombocytopenia (75%), nausea and vomiting (61%), liver toxicity (46%), infection/inflammation/leukocytosis (19%), nephrotoxicity (7%), bleeding (7%), and capillary leak (4%). Most SAEs were grade one or two only; 5% were grade three or four requiring additional interventions. Of note, one patient suffered a cardiac arrest during his first CS-PHP session; as a consequence, the patient was removed from the subsequent analysis and instead was treated via selective internal radiation therapy (SIRT) after successful treatment of a right coronary artery occlusion. Detailed SAE data per patient and procedure are summarized in Tables 2 and 3, respectively.

**Table 2:** Serious adverse events (SAE) during and after chemosaturation with percutaneous hepatic perfusion per procedure

per procedure	grade 1		grade 2		grade 1&2^a^		grade 3		grade 4		grade 3&4^a^	
anemia	12	43%	11	39%	23	82%	4	14%	0	0%	4	14%
Leukopenia	19	68%	4	14%	23	82%	4	14%	0	0%	4	14%
Thrombocytopenia	17	61%	0	0%	17	61%	4	14%	0	0%	4	14%
Liver toxicity	13	46%	0	0%	13	46%	0	0%	0	0%	0	0%
Nephrotoxicity	2	7%	0	0%	2	7%	0	0%	0	0%	0	0%
Vascular compl./bleeding	2	7%	0	0%	2	7%	0	0%	0	0%	0	0%
Nausea/vomiting	2	7%	15	54%	17	61%	0	0%	0	0%	0	0%
Cardiovascular	0	0%	0	0%	0	0%	0	0%	1	4%	1	4%
Infection/inflammation	5	18%	0	0%	5	18%	0	0%	0	0%	0	0%
Capillary leak	0	0%	1	4%	1	4%	0	0%	0	0%	0	0%

Of note: grading of SAE was based on Common Terminology Criteria for Adverse Events (CTCAE) Version 5.0 (2017). ^a^Combined number of grade 1 and grade 2 SAEs or grade 3 and grade 4 SAEs, respectively

**Table 3:** Serious adverse events (SAE) during and after chemosaturation with percutaneous hepatic perfusion per patient

*per patient*	grade 1		grade 2		grade 1/2^a^		grade 3		grade 4		grade 3/4	
Anemia	6	21%	7	25%	13	46%	3	11%	0	0%	3	11%
leukopenia	9	32%	3	11%	12	43%	4	14%	0	0%	4	14%
Thrombocytopenia	9	32%	0	0%	9	32%	4	14%	0	0%	4	14%
Liver toxicity	10	36%	0	0%	10	36%	0	0%	0	0%	0	0%
Nephrotoxicity	2	7%	0	0%	2	7%	0	0%	0	0%	0	0%
Vascular compl./bleeding	2	7%	0	0%	2	7%	0	0%	0	0%	0	0%
Nausea/vomiting	2	7%	11	39%	13	46%	0	0%	0	0%	0	0%
Cardiovascular	0	0%	0	0%	0	0%	0	0%	1	4%	1	4%
Infection/inflammation	5	18%	0	0%	5	18%	0	0%	0	0%	0	0%
Capillary leak	0	0%	1	4%	1	4%	0	0%	0	0%	0	0%

Of Note: Grading of SAE was based on Common Terminology Criteria for Adverse Events (CTCAE) Version 5.0 (2017). ^a^Combined number of grade 1 and grade 2 SAEs or grade 3 and grade 4 SAEs, respectively. This table takes into account that several patients received more than one Chemosaturation with percutaneous hepatic perfusion

## Discussion

The results of this investigation demonstrate that CS-PHP is a safe and efficacious method of treating liver-dominant metastatic uveal melanoma. After 28 CS-PHP treatments in 16 patients, the observed rates of progression-free and overall survival of 11.1 months and 27.4 months, respectively, were substantially higher than the progression-free survival of 3.3 months and a median overall survival of 10.2 months for conventional chemotherapy reported in a recent meta-analysis [1, 6]. Additionally, these results are more favorable than those reported in a prior study by Vogel et al. that included patients with metastatic disease limited to the liver, which reported a median overall survival of 9.6 months and a median progression-free survival of 12.4 months [14]. However, this prior work was a retrospective analysis of data acquired from multiple centers throughout Germany, resulting in a substantially higher heterogenicity of data and a less consistent postinterventional standard of care [14].

In contrast, half of all patients included in this study had also extrahepatic metastatic uveal melanoma. Sub-analysis of patients with extrahepatic manifestations before their first CS-PHP demonstrated that these patients also had a superior OS compared to work of Vogel et al. and the meta-analyses of Yang et al. and Khoja et al. [1, 6, 14]. Consequently, these new results indicate that CS-PHP is also an appropriate treatment for patients presenting with liver-dominant metastatic disease.

Importantly, comorbidities must be taken into account when considering CS-PHP as a treatment approach, since serious adverse events were observed in most patients. The most common of these SAEs were anemia, leukopenia, and thrombocytopenia. Fortunately, grade three and grade four SAEs, which by definition necessitate the need for additional treatment, were observed only in a small number of patients. The numbers reported in this study are in line with those observed in prior work [1, 14, 20]. However, it is particularly important to recognize that CS-PHP causes substantial stress on the cardiovascular system. All patients experienced significant hypotension when extracorporeal filtration was initially established. These effects were countered by the administration of large volumes of saline and colloid solutions (up to eight liters) as well as high doses of catecholamines, interventions requiring the additional anesthesia expertise. Due to this cardiovascular challenge, one patient suffered a cardiac arrest during the procedure; CS-PHP was discontinued, and further medical treatment revealed a right coronary artery occlusion, which was successfully treated. Retrospectively, the patient’s preprocedural staging CT showed signs of coronary artery disease. As a consequence, subsequent patients received more thorough screening for the presence of cardiovascular risk factors. For those patients deemed to be at high cardiovascular risk due to coronary artery disease, pre-treatment of the heart or alternative therapies (e.g., SIRT) would have been recommended.

Limitations of this work include the study’s retrospective design and limited sample size due to its inclusion of only a single center. While uveal melanoma is the most common intraocular malignancy, it is a relatively rare disease overall, and larger patient numbers are likely to be achieved only by the use of pooled data analysis from multiple centers, an approach that is limited in retrospective studies due to the inconsistency of treatment regimens and post-procedural patient care. As a result, a single center was used to minimize these confounding effects at the cost of a more limited sample size. This study included data collected from 28 CS-PHP procedures performed on 16 patients; consequently, some patients received multiple procedures, resulting in superior survival. A further limitation of the study is the short median SAE-related follow-up after CS-PHP of 16 days; melphalan related side effects may occur as late as 14 days after application [21]. Hence, some SAEs may have been missed due to short follow-up times in several patients.

## Conclusion

CS-PHP is a safe and efficacious treatment modality for liver-dominant metastatic uveal melanoma. The observed rates of overall and progression-free survival exceeded the reported outcomes of systemic treatment. SAEs were frequent, with most limited to grades one and two and not requiring additional intervention. However, care must be taken in patients with suspected coronary artery disease due to therapy-related strain on the cardiovascular system.

## Data Availability

Source data tables are available.

## Abbreviations

ACT: Activated clotting time
BMI: Body mass index
CI: Confidence interval
CS-PHP: Chemosaturation with percutaneous hepatic perfusion
CT: Computed tomography
CTCAE: Common Terminology Criteria for Adverse Events
MRI: Magnetic resonance imaging
OS: Overall survival
PD: Progressive disease
PFS: Progression free survival
PR: Partial remission
SAE: Serious adverse events
SD: Stable disease
SIRT: Selective internal radiation therapy
